# Expression of a fungal exo-β-1,3-galactanase in Arabidopsis reveals a role of type II arabinogalactans in the regulation of cell shape

**DOI:** 10.1093/jxb/eraa236

**Published:** 2020-05-29

**Authors:** Yoshihisa Yoshimi, Katsuya Hara, Mami Yoshimura, Nobukazu Tanaka, Takumi Higaki, Yoichi Tsumuraya, Toshihisa Kotake

**Affiliations:** 1 Division of Life Science, Graduate School of Science and Engineering, Saitama University, Shimo-okubo, Sakura-ku, Saitama, Japan; 2 Department of Biochemistry, University of Cambridge, Cambridge, UK; 3 Program of Biotechnology, Graduate School of Integrated Sciences for Life, Hiroshima University, Kagamiyama, Higashi-Hiroshima, Hiroshima, Japan; 4 Department of Gene Science, Integrated Experiment Support/Research Division, Natural Science Center for Basic Research and Development, Higashi-Hiroshima, Hiroshima, Japan; 5 International Research Organization for Advanced Science and Technology, Kumamoto University, Kurokami, Chuo-ku, Kumamoto, Japan; 6 Green Biology Research Center, Saitama University, Shimo-okubo, Sakura-ku, Saitama, Japan; 7 University of Manchester, UK

**Keywords:** Arabidopsis, arabinogalactan-protein, exo-β-1, 3-galactanase, extracellular proteoglycan, fungal enzyme, inducible expression system, *Irpex lacteus*, tissue disorganization, type II arabinogalactan

## Abstract

Arabinogalactan-proteins (AGPs) are a family of plant extracellular proteoglycans implicated in many physiological events. AGP is decorated with type II arabinogalactans (AGs) consisting of a β-1,3-galactan backbone and β-1,6-galactan side chains, to which other sugars are attached. Based on the fact that a type II AG-specific inhibitor, β-Yariv reagent, perturbs growth and development, it has been proposed that type II AGs participate in the regulation of cell shape and tissue organization. However, the mechanisms by which type II AGs participate have not yet been established. Here, we describe a novel system that causes specific degradation of type II AGs in Arabidopsis, by which a gene encoding a fungal exo-β-1,3-galactanase that specifically hydrolyzes β-1,3-galactan backbones of type II AGs is expressed under the control of a dexamethasone-inducible promoter. Dexamethasone treatment increased the galactanase activity, leading to a decrease in Yariv reagent-reactive AGPs in transgenic Arabidopsis. We detected the typical oligosaccharides released from type II AGs by Il3GAL in the soluble fraction, demonstrating that Il3GAL acted on type II AG in the transgenic plants. Additionally, this resulted in severe tissue disorganization in the hypocotyl and cotyledons, suggesting that the degradation of type II AGs affected the regulation of cell shape.

## Teaser

We developed a new system that causes specific degradation of type II arabinogalactans by dexamethasone-inducible expression of a fungal exo-β-1,3-galactanase in Arabidopsis, producing severe tissue disorganization in the hypocotyl and cotyledons.

## Introduction

Type II arabinogalactans (AGs) are plant-specific glycans generally found as the carbohydrate component of the extracellular proteoglycans called arabinogalactan-proteins (AGPs). In several plants, they have also been detected as side chains of pectin rhamnogalacturonan I and as a secreted polysaccharide in cell walls (Ponder and Richards, 1997; Mohnen, 2008; Caffall and Mohnen, 2009). Although the structures of type II AGs are diverse, depending on plant species, tissue, and developmental stage (Tsumuraya *et al.*, 1988), they universally consist of a β-1,3-galactan backbone and β-1,6-galactan side chains, to which l-Ara and other additional sugars such as GlcA, 4-*O*-methyl-GlcA (4-*O*-Me-GlcA), and l-Fuc are attached (Tryfona *et al.*, 2012; Tan *et al.*, 2013). These features distinguish type II AGs from type I AGs which have a β-1,4-galactan backbone (Mohnen, 2008). Because type II AGs can be detected in angiosperms, gymnosperms, ferns, mosses, and liverworts, they have been expected to have important functions throughout the plant kingdom (Jermyn and Yeow, 1975; Clarke *et al.*, 1978).

The participation of type II AGs in various physiological processes has been shown in studies using a specific inhibitor, β-Yariv reagent, which is a phenylazo compound with β-glucosyl or β-galactosyl residues at its termini specifically binding to β-1,3-galactan (Yariv *et al.*, 1962; Kitazawa *et al.*, 2013). In tobacco (*Nicotiana tabacum*) suspension-cultured cells, treatment with β-Yariv reagent causes extreme cell bulging together with disorganization and depolymerization of cortical microtubules (CMTs) (Sardar *et al.*, 2006). It also strongly inhibits apical cell expansion in the moss *Physcomitrella patens* (Lee *et al.*, 2005). On the other hand, this does not exclude the possibility that the phenotypes observed in plants treated with β-Yariv reagent are caused, at least in part, by secondary effects of this phenylazo compound, because β-Yariv reagent forms an insoluble precipitate with type II AGs and AGPs in cell walls (Olmos *et al.*, 2017).

Several Arabidopsis (*Arabidopsis thaliana*) AGP mutants exhibit altered cell shape and tissue disorganization. The *salt-overly sensitive5* (*sos5*)*/fasciclin-like agp4* (*fla4*) mutant, which has a defect in an AGP core protein with fasciclin-like domains, shows root swelling and reduced root growth when treated with a high concentration of NaCl, while it does not show these phenotypes under normal conditions (Shi *et al.*, 2003). Nevertheless, with >80 AGP genes in Arabidopsis, gene redundancy makes it difficult to understand the physiological functions and importance of AGPs based on the study of Arabidopsis knockout lines (Borner *et al.*, 2003; Ma *et al.*, 2017).

It is probable that type II AGs contain physiologically important ‘epitopes’ to participate in various physiological events in plants. In *Torenia foumieri*, 4-*O*-Me-GlcA-1,6-Gal (MeGlcAGal), a non-reducing terminal structure of type II AGs, has been shown to function as a signaling factor during fertilization (Mizukami *et al.*, 2016). This signaling factor, called AMOR, confers on pollen tubes the competency to respond to ovular attractant LURE peptides for pollen tube guidance. However, since type II AGs are widely observed in other tissues and organs including the hypocotyl and roots in plants, it is likely that AGs have other distinct biological functions.

As a novel approach in the investigation of the physiological importance and exploration of molecular functions of AGPs, we developed a dexamethasone (Dex)-inducible system for *in vivo* specific degradation of AG sugar chains using exo-β-1,3-galactanase, a fungal enzyme that causes drastic degradation of type II AGs by hydrolyzing their β-1,3-galactan backbones (Tsumuraya *et al.*, 1990; Kotake *et al.*, 2009). With this system, we observed severe tissue disorganization in the hypocotyl and cotyledons of transgenic Arabidopsis when the degradation of type II AG was induced by treatment with Dex (Aoyama and Chua, 1997), indicating that type II AG plays an essential role in cell morphology.

## Materials and methods

### Materials

The β-1,3-galactan substrate for exo-β-1,3-galactanase from *Ilpex lacteus* (Il3GAL) was prepared from gum arabic (*Acacia senegal*; Sigma-Aldrich Japan, Tokyo, Japan) by Smith degradation as described previously (Goldstein *et al.*, 1956; Kitazawa *et al.*, 2013). Radish root and leaf AGPs were extracted and purified from radish (*Raphanus sativus* L.) as described previously (Shimoda *et al.*, 2014; Inaba *et al.*, 2015). The polyclonal antibody raised against a partial sequence of Il3GAL, GGGDQTYSYTDTKI, was purchased from Eurofins Genomics (Tokyo, Japan). The polyclonal antibody serum from rabbit was subjected to ammonium sulfate precipitation, and the 20–40% saturated fraction was used for western blotting.

### Preparation of recombinant Il3GAL and point-mutated rIl3GAL

*Il3GAL* (accession number AB461394) was cloned in a previous study (Kotake *et al.*, 2009). To generate the point-mutated *Il3GAL* (*Il3GAL-PM*) gene, the point mutation E102A that replaces the conserved Glu with Ala was introduced by PCR using the primers Il3GAL-PM-F and Il3GAL-PM-R (see Supplementary Table S1 at *JXB* online). For expression in *Pichia pastoris*, the *Il3GAL* and *Il3GAL-PM* fragments were subcloned into pPICZαC plasmid (Invitrogen, Waltham, MA, USA). The methylotrophic *P. pastoris* (*Pichia* yeast) strain KM71H (Invitrogen) was then transformed by electroporation with the constructs, Il3GAL/pPICZαC or Il3GAL-PM/pPICZαC. The transformants were selected based on zeocine resistance and cultured in YPG medium containing 1% (w/v) yeast extract, 2% (w/v) peptone, and 1% (w/v) glycerol at 30 °C with shaking at 90 rpm for 2 d. The cells were collected by centrifugation at 1500 *g* for 5 min, washed with ice-cold water, and suspended in 50 ml of YP medium containing 1% (w/v) yeast extract and 2% (w/v) peptone. The expression of recombinant enzymes was induced by 1% (v/v) methanol, and the yeast was cultured for another 5 d with addition of 1% (v/v) methanol each day. After centrifugation at 1500 *g* for 5 min, the supernatant was collected as crude enzyme. The recombinant Il3GAL (rIl3GAL) and recombinant Il3GAL-PM (rIl3GAL-PM) were purified from crude enzyme solution by size exclusion chromatography and ion-exchange chromatography as described previously (Kotake *et al.*, 2009). The purity of rIl3GAL and rIl3GAL-PM was examined by SDS–PAGE (Laemmli, 1970).

### Generation of transgenic plants and Dex treatment

Arabidopsis ecotype Columbia (Col-0) was used in the present study. The cDNA for the *Il3GAL* or *Il3GAL-PM* sequence without the part corresponding to its signal peptide was PCR amplified using KOD2 DNA polymerase (Toyobo, Tokyo, Japan) and the set of primers, Il3GAL-F+*Bam*HI and Il3GAL-R+*Spe*I (Supplementary Table S1) (Kotake *et al.*, 2009). The cDNA sequence corresponding to the signal sequence of Arabidopsis AGP4 (At5g10430) was PCR amplified with the primers AtAGP4-F+*Xho*I and AtAGP4-R+*Bam*HI (Supplementary Table S1). These two fragments were fused using the *Bam*HI site. The resulting fragment, AGP4 signal sequence–Il3GAL, was subcloned between the *Xho*I and *Spe*I sites of pTA7001 (Aoyama and Chua, 1997). To generate *Dex::Il3GAL* and *Dex::Il3GAL-PM* plants, the gene constructs were introduced into Arabidopsis by agrobacterium-mediated (*Rhizobium radiobacter*, EHA105 strain) transformation (Clough and Bent, 1998). Transformants were selected using Murashige and Skoog (MS) medium (Murashige and Skoog, 1962) containing 20 µg ml^–1^ hygromycin B and 50 µg ml^–1^ carbenicillin. The hygromycin B-resistant seedlings were isolated as individual transgenic lines. For the present study, five independent transgenic lines (#1, 2, 3, 4, and 5) for *Dex::Il3GAL* plants and nine independent transgenic lines (#1, 2, 3, 4, 5, 6, 7, 8, and 9) for *Dex::Il3GAL-PM* plants were obtained. Homozygous plants of the T_3_ generation were used in all experiments. Plants were grown at 22 °C under continuous light. For the analysis of galactanase activity, the plants were first grown on MS agar medium for 2 weeks, then treated with a solution containing 10 μM Dex and 0.1% DMSO for 2 d. As the negative control, plants were treated with 0.1% DMSO solution. To observe phenotypes, Arabidopsis seeds were sown on MS agar medium containing 0.1, 1, or 10 μM Dex, and the plants were grown for a week.

### Semi-quantitative analysis of Il3GAL mRNAs

The relative expression level of *Il3GAL* in *Dex::Il3GAL* plants was estimated by semi-quantitative reverse transcription–PCR (RT–PCR). Total RNA was extracted from Arabidopsis seedlings treated with Dex for 0.5, 1, 3, 6, 12, or 24 h with an Isogen kit (Nippon Gene, Tokyo, Japan). Single-stranded cDNA was synthesized from total RNA using oligo(dT)_12–18_ primer. A set of specific primers, Il3GAL-QPCR-F and Il3GAL-QPCR-R (Supplementary Table S1), for *Il3GAL* was designed using the Primer3 program (http://bioinfo.ut.ee/primer3-0.4.0/). As the internal control, the expression level of *ACTIN2* (At3g18780) was measured with a set of specific primers, ACT2-RTP-F and ACT2-RTP-R (Supplementary Table S1). The PCR was performed with a SYBR Premix Ex Taq kit (Takara Bio Inc., Otsu, Japan) under the following conditions: 10 s denaturing at 95 °C, 30 s annealing at 60 °C, and 20 s amplification at 72 °C, 40 cycles.

### Measurement of galactanase activity in transgenic plants

The galactanase activity of rIl3GAL and rIl3GAL-PM was measured with a reaction mixture containing 1 mg ml^–1^ β-1,3-galactan prepared from gum arabic, 50 mM sodium acetate buffer (pH 5.0), and rIl3GAL and rIl3GAL-PM at 37 °C. The reducing sugars were measured colorimetrically by the neocuproine method (Dygert *et al.*, 1965). One unit of enzyme activity is capable of producing 1 µmol of reducing power from the substrate per minute.

To measure the galactanase activity in the transgenic plants, the seedlings of *Dex::Il3GAL* and *Dex::Il3GAL-PM* plants treated with 10 μM Dex were ground in 20 mM sodium acetate buffer (pH 5.0) with a mortar and pestle and centrifuged at 10 000 *g*. The resulting supernatant was collected as the soluble fraction. The pellet was resuspended in the same buffer containing 1 M NaCl at 4 °C for 1 h and then centrifuged. The resulting supernatant was collected as the cell wall-bound fraction. The galactanase activity of soluble and cell wall-bound fractions was measured using 1 mg ml^–1^ β-1,3-galactan as substrate as described above.

### Western blotting

Il3GAL and Il3GAL-PM proteins induced by treatment with Dex in the transgenic Arabidopsis were detected by western blotting. As Il3GAL and Il3GAL-PM fused with the signal peptide of Arabidopsis AGP4 are designed to be secreted to cell walls in the transgenic plants, these proteins can be collected in the soluble fraction. Two-week-old plants treated with 10 μM Dex and 0.1% DMSO for 2 d were ground with a pestle in 20 mM sodium acetate buffer (pH 5.0). After centrifugation at 10 000 *g*, the supernatant was collected and subjected to SDS–PAGE. The Il3GAL protein in the gel was transferred to a polyvinylidene difluoride membrane and then detected with anti-Il3GAL antibody (rabbit IgG) as the primary antibody (1:2000 dilution) and a horseradish peroxidase-conjugated anti-rabbit IgG antibody (GE Healthcare Life Sciences, Chicago, IL, USA) as the secondary antibody (1:10 000 dilution). Chemi Doc XRS (Bio-Rad, Tokyo, Japan) was used to detect chemiluminescence.

### Quantification of type II AGs

Plants treated with Dex were ground in 50 mM MOPS-KOH buffer (pH 6.5) and boiled for 30 min. After centrifugation, the supernatant was collected as the hot water (HW)-soluble fraction. The amount of type II AGs in the HW-soluble fraction was determined by radial gel diffusion assay with a gel containing 40 µg ml^–1^ β-Gal-Yariv, 150 mM sodium chloride, 0.02% (w/v) sodium azide, and 1% (w/v) agarose (van Holst and Clarke, 1985).

### Detection and identification of oligosaccharides

For the analysis of action of recombinant enzymes expressed in *Pichia* yeast, rIl3GAL and rIl3GAL-PM were reacted with 1 mg ml^–1^ AGPs from radish leaves in 50 mM sodium acetate buffer (pH 5.0) at 37 °C. Released Gal and oligosaccharides were derivatized with *p*-aminobenzoic acid ethyl ester (ABEE; Wako, Osaka, Japan) (Matsuura and Imaoka, 1988), and detected by HPLC as previously described (Yoshimi *et al.*, 2017). ABEE-labeled isomaltooligosaccharides (IMOs) were used as molecular makers for the elution positions (Konishi *et al.*, 2007).

For the identification of type II AG-derived oligosaccharides in the transgenic plants, the soluble fraction was prepared from 230–500 mg of *Dex::Il3GAL* and *Dex::Il3GAL-PM* plants grown on MS agar medium containing 10 μM Dex. Oligosaccharides included in the soluble fraction were labeled with ABEE and detected by HPLC as described above. ABEE-labeled oligosaccharides were further digested with α-l-arabinofuranosidase from *Aspergillus niger* (Arafase) from Megazyme and/or recombinant β-glucuronidase from *Aspergillus niger* expressed in *P. pastoris* (GlcAase) as described previously (Konishi *et al.*, 2008).

The standard oligosaccharides were prepared and purified as previously described (Shimoda *et al.*, 2014). AGPs from radish roots were first hydrolyzed into oligosaccharides with 80 mU of rIl3GAL and 80 mU of recombinant endo-β-1,3-galactanase from *Neurospora crassa* (rNcEn3GAL), which were expressed in *P. pastoris*, at 37 °C overnight (Yoshimi *et al.*, 2017). The resulting hydrolysate including oligosaccharides was applied to a Sephadex G-100 column to remove polysaccharides. The oligosaccharides were fractionated into neutral and acidic oligosaccharide fractions by anion exchange chromatography using a DEAE-cellulose (SERVA, Heidelberg, Germany) column (HCO_3_^–^, 2.2×8 cm). The neutral and acidic fractions were desalted by DOWEX 50Wx8 (Dow Chemical Company, Midland, MI, USA) and lyophilized.

### Preparation of cross-sections and observation by SEM

Preparation of cross-sections was performed as described previously with minor modifications (Nitta *et al.*, 2009; Abedi *et al.*, 2018). Briefly, 7-day-old seedlings treated with Dex were pre-fixed in 2% glutaraldehyde in 0.1 M sodium phosphate buffer (pH 7.4) at room temperature for 2 h and at 4 °C overnight. After rinsing in the buffer, seedlings were post-fixed in the buffer containing 2% OsO_4_ on ice for 2 h and then dehydrated in a graded ethanol series. The post-fixed samples were substituted with an ethanol:propylene oxide mixture (1:1, v/v) once and with propylene oxide twice, then embedded in EPOK 812 resin (Oken Shoji, Tokyo, Japan). Semi ultra-thin sections (~500 nm in thickness) were cut with a glass knife on an Ultracut N ultramicrotome (Reichert Technologies, Depew, NY, USA) and stained by dipping in 0.01% (w/v) toluidine blue O solution for a minute followed by gentle removal of the stain.

For SEM observation, pre-fixed samples were lyophilized with a FDC10 freezer and FD6510 freeze dryer (SUN Technologies, Alpharetta GA, USA), and sputter-coated with platinum. The samples were visualized using a JSM 5610 scanning elctron microscope (JEOL, Tokyo, Japan) at an accelerating voltage of 5 kV.

## Results

### Properties of exo-β-1,3-galactanase, Il3GAL, and its point-mutated form *in vitro*

**Fig. 1.**
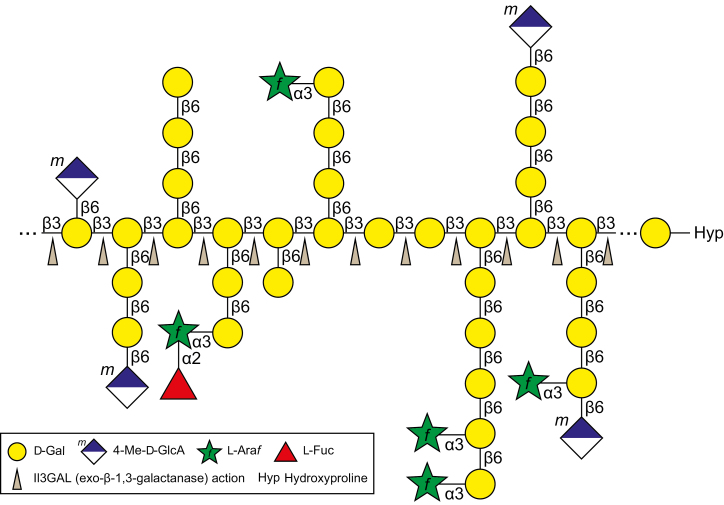
Model structure of type II AG and action of Il3GAL. A model structure of type II AG is shown. Il3GAL, represented by an arrowhead, hydrolyzes β-1,3-galactan backbones irrespective of β-1,6-galactan side chains from non-reducing terminal residues, which results in drastic breakdown of type II AGs, releasing various oligosaccharides derived from the side chains. l-Ara*f*, l-arabinofuranose.

**Fig. 2.**
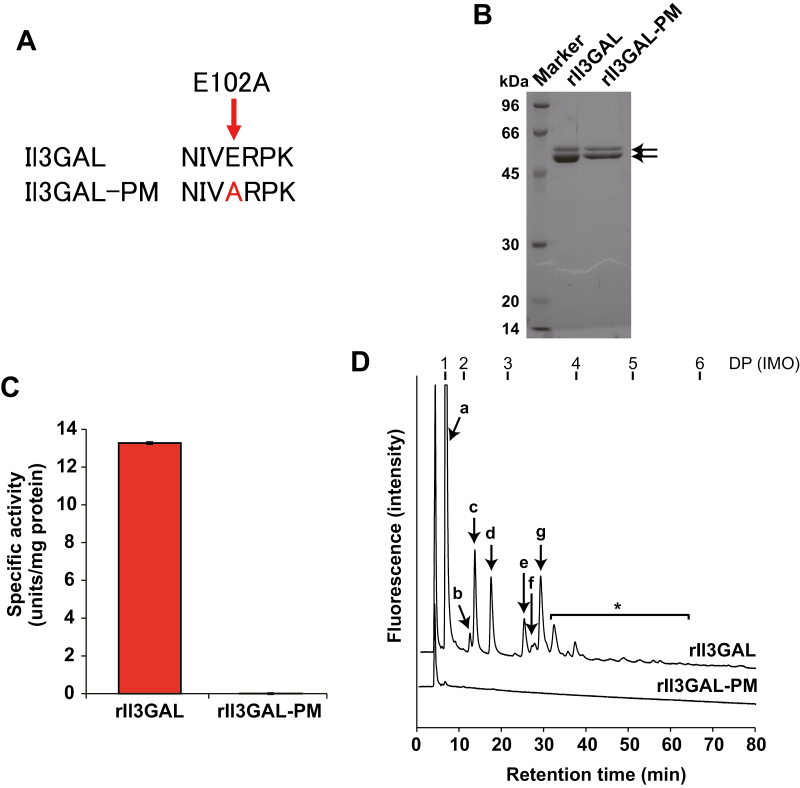
Enzymatic activity of rIl3GAL and rIl3GAL-PM. (A) Point mutation in the catalytic site of Il3GAL. The Glu residue was replaced with Ala to generate rIl3GAL-PM. (B) The purity of recombinant proteins. The rIl3GAL and rIl3GAL-PM proteins were examined by SDS–PAGE. Proteins in the gel were stained with Coomassie Brilliant Blue R-250. Arrows indicate rIl3GAL and rIl3GAL-PM. (C) Galactanase activity. The activity of recombinant enzymes was measured using β-1,3-galactan as substrate. Data are mean values ±SD (*n*=3 technical replicates). (D) Released oligosaccharides from radish leaf AGPs by the action of rIl3GAL or rIl3GAL-PM. Enzymatic hydrolysis products were derivatized with ABEE and detected by HPLC. Arrows indicate oligosaccharides as follows: a, Gal; b, MeGlcAGal; c, Gal_2_; d, l-Ara-β-1,6-Gal_2_; e, MeGlcAGal_2_; f, Gal_3_; g, AraGal_3_. Asterisks indicate oligosaccharides released from type II AGs but not assigned. The elution positions of glucose and IMOs with degree of polymerization (DP) 2–6 are indicated on the top.

Although their structures are diverse and heterogeneous, almost all type II AGs have a β-1,3-galactan backbone in common. Exo-β-1,3-galactanase, Il3GAL, is a fungal enzyme from *I. lacteus*, which specifically hydrolyzes the β-1,3-galactan backbones of type II AGs (Tsumuraya *et al.*, 1990; Kotake *et al.*, 2009). Il3GAL differs from plant endogenous enzymes such as β-galactosidase and α-l-arabinofuranosidase (Kotake et al., 2005, 2006), as it causes drastic degradation of type II AGs by hydrolyzing the β-1,3-galactan backbone irrespective of the β-1,6-galactan side chains (Fig. 1). As the result of action by Il3GAL, type II AGs are hydrolyzed into oligosaccharides derived from the β-1,6-galactan side chains (Tsumuraya *et al.*, 1990; Tryfona *et al.*, 2012; Inaba *et al.*, 2015). In the present study, *in vivo* specific degradation by Il3GAL introduced into Arabidopsis was examined. Since, however, it was conceivable that the presence of Il3GAL itself generated stress in the plants, because Il3GAL is a fungal protein, we compared the effect with a negative control, Il3GAL-PM, which has the mutation E102A at its conserved residue (Fig. 2A) (Ichinose *et al.*, 2005; Kotake *et al.*, 2009). To confirm the loss of activity of Il3GAL-PM, rIl3GAL and rIl3GAL-PM were prepared by expression in *Pichia* yeast (Fig. 2B). The rIl3GAL expressed in *Pichia* yeast appeared as two bands with relative molecular masses of 54 kDa and 49 kDa on SDS–PAGE, which is larger than that (45 520 Da) predicted from the amino acid sequence. This can be explained as we have shown in a previous study that the apparent molecular mass of rIl3GAL shifts to 45 kDa after removal of *N*-glycans, and we conclude that the protein bands observed here are indeed rIl3GAL in different states of *N*-glycosylation (Gemmill and Trimble, 1999; Kotake *et al.*, 2009; Strasser,S 2016)

While rIl3GAL exhibited strong hydrolytic activity (13.3 U mg^–1^ protein) towards β-1,3-galactans, releasing various β-1,6-galactooligosaccharides derived from side chains from radish leaf AGPs, rIl3GAL-PM did not show measurable activity (<0.001 U mg^–1^ protein), resulting in no released oligosaccharide from radish leaf AGPs *in vitro* (Fig. 2C, D).

### *In vivo* degradation of type II AGs by means of a fungal enzyme

Using the *Il3GAL* gene, we accomplished the specific degradation of type II AGs *in vivo* under the control of a Dex-inducible promoter in Arabidopsis. To ensure the secretion of Il3GAL *in muro*, the signal sequence of Il3GAL was replaced with that of Arabidopsis AGP4 (Supplementary Fig. S1). We designated the transgenic Arabidopsis *Dex::Il3GAL*.

**Fig. 3.**
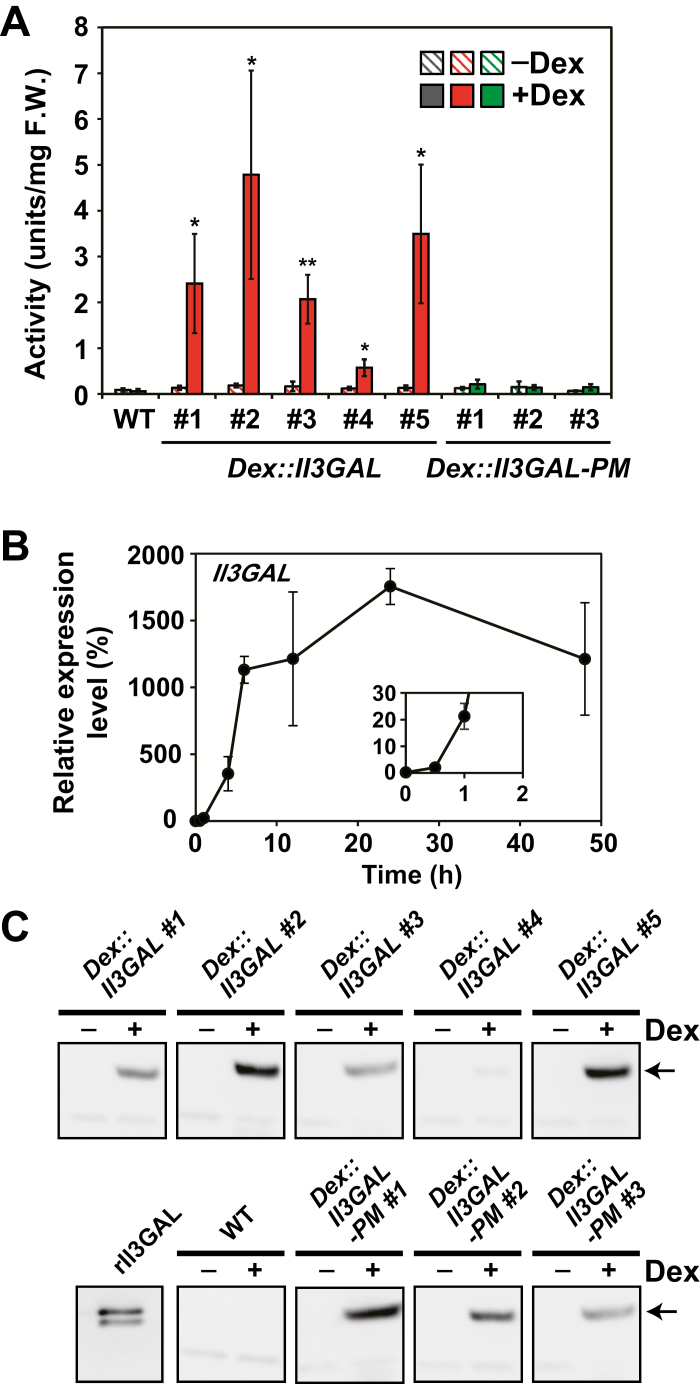
*Il3GAL* expression induced by Dex *in planta*. (A) Galactanase activity in *Dex::Il3GAL* plants treated with Dex. *Dex::Il3GAL* and *Dex::Il3GAL-PM* plants were first grown in the absence of Dex for 2 weeks and then treated with 10 µM Dex for 2 d. The negative controls were treated with 0.1% DMSO solution for 2 d. Values indicate the total activity obtained from the soluble and wall-bound fractions. F.W., fresh weight. Data are mean values ±SD (*n*=3 biological replicates). Asterisks indicate significant differences between plants with and without Dex treatment (Student’s *t*-test, *P<0.05; **P<0.01). (B) Induction of the *Il3GAL* gene. *Dex::Il3GAL* #2 plants grown in the absence of Dex were treated with 10 µM Dex for 0.5, 1, 3, 6, 12, 24, and 48 h. The expression level of *Il3GAL* was determined by semi-quantitative RT–PCR. The values are shown as expression levels relative to *ACTIN2*. Data are mean values ±SD (*n*=3 biological replicates). (C) Detection of Il3GAL and Il3GAL-PM. Il3GAL and Il3GAL-PM proteins in the soluble fraction were detected by western blotting using anti-Il3GAL antibody. The arrow indicates Il3GAL or Il3GAL-PM protein.

In *Dex::Il3GAL* plants, the galactanase activity toward β-1,3-galactan rapidly increased under treatment in a dose-dependent manner, whereas no increase was observed in the wild type (WT) (Fig. 3A; Supplementary Fig. S2), indicating that galactanase activity could be regulated by Dex concentration. In fact, the expression of the *Il3GAL* gene occurred within 1 h after Dex treatment and reached a maximum at 24 h (Fig. 3B). Western blot analysis using antibody raised against an Il3GAL peptide also confirmed that Il3GAL protein was produced by Dex treatment in the transgenic plants (Fig. 3C).

On the other hand, in *Dex::Il3GAL-PM* transgenic plants, galactanase activity was almost unchanged from that of the WT, and, as was expected, they did not show increased activity after Dex treatment (Fig. 3A). The weak activity observed probably resulted from endogenous β-galactosidases (Kotake *et al.*, 2005). Accumulation of Il3GAL and Il3GAL-PM proteins was confirmed immunologically, indicating that *Dex::Il3GAL-PM* plants were suitable as a negative control (Fig. 3C).

### Degradation of AGP in transgenic plants

**Fig. 4.**
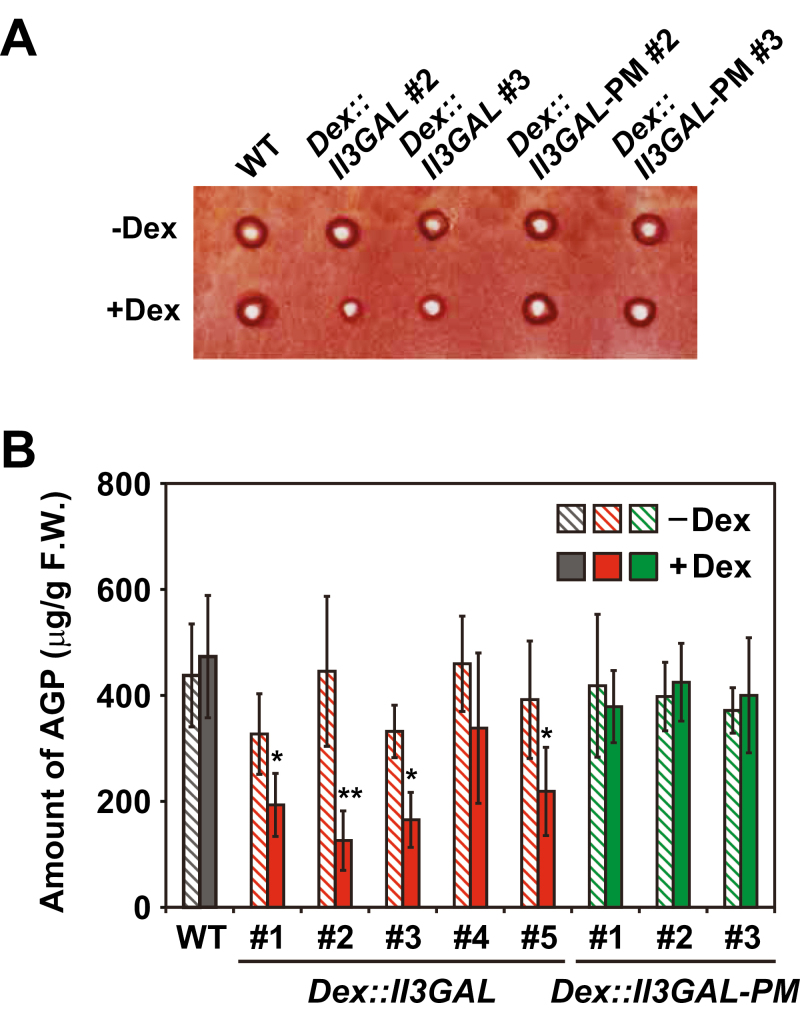
Degradation of type II AGs *in muro*. (A) β-Yariv-reactive type II AGs in the transgenic plants. A radial gel diffusion assay with β-Yariv reagent was performed with the soluble fraction. Representative results for WT, *Dex::Il3GAL* #2 and #3, and *Dex::Il3GAL-PM* #2 and #3 are shown here. (B) Estimated amount of type II AGs. The area of the halo formed by β-Yariv-reactive type II AGs was calculated and plotted. Data are mean values ±SD (*n*=3 biological replicates). Asterisks indicate significant differences between amounts with and without Dex treatment (Student’s *t*-test, *P<0.05; **P<0.01).

To evaluate the degree of type II AG degradation, a radial gel diffusion assay with β-Yariv reagent was performed. As β-Yariv reagent binds to any β-1,3-galactan backbone that is larger than β-1,3-galactopentaose (Kitazawa *et al.*, 2013), β-Yariv reactivity can be used to estimate the amount of β-1,3-galactans in the samples. The soluble fraction, which includes most AGPs, was prepared from *Dex::Il3GAL* plants and subjected to Yariv assay (Fig. 4A). In *Dex::Il3GAL* plants #1 and #2 grown in the presence of Dex, the amount of β-Yariv reagent-reactive (β-Yariv-reactive) AGPs was drastically reduced (42% and 72%, respectively, were lost compared with the amount in plants grown in the absence of Dex), whereas *Dex::Il3GAL-PM* and WT plants did not show a significant change under Dex treatment (Fig. 4B).

**Fig. 5.**
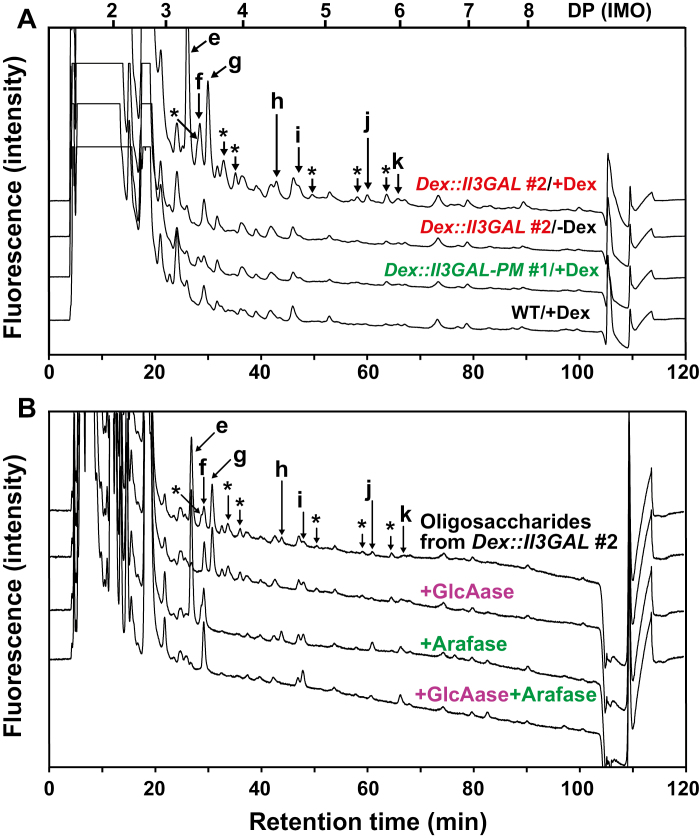
Identification of oligosaccharides liberated from type II AGs *in vivo*. (A) Chromatogram of detected oligosaccharides liberated from type II AGs by the action of Il3GAL. The oligosaccharides in the soluble fraction were derivatized with ABEE and detected by HPLC. Arrows indicate liberated oligosaccharides as follows: e, MeGlcAGal_2_; f, Gal_3_; g, AraGal_3_; h, MeGlcAGal_3_; i, Gal_4_; j, MeGlcA-β-1,6-Gal_4_; k, β-1,6-galactopentaose. Asterisks indicate oligosaccharides released from type II AGs but not assigned. The elution positions of glucose and IMOs with DP 2–8 are indicated on the top. (B) Enzymatic digestion of AG oligosaccharides. The labeled oligosaccharides were digested with GlcAase (+GlcAase), Arafase (+Arafase), or both enzymes (+GlcAase +Arafase). The elution positions of β-1,6-galactooligosaccharides and MeGlcA-β-1,6-galactooligosaccharides are shown in Supplementary Fig. S3.

While plant endogenous glycoside hydrolases including β-galactosidase and α-l-arabinofuranosidase only act on non-reducing terminal residues of type II AGs releasing monosaccharides, Il3GAL causes dramatic degradation by directly hydrolyzing the β-1,3-galactan backbone, which releases the oligosaccharides from side chains. In order to confirm that the enzyme functioned in transgenic plants, we measured oligosaccharides by HPLC. In the soluble fraction of *Dex::Il3GAL* plants treated with Dex, we identified an accumulation of specific oligosaccharides typical for type II AGs, such as β-1,6-galactotriose (Gal_3_), 4-*O*-Me-GlcA-β-1,6-Gal_2_ (MeGlcAGal_2_), l-arabinosyl-Gal_3_ (AraGal_3_), and 4-*O*-Me-GlcA-β-1,6-Gal_3_ (MeGlcAGal_3_) (Fig. 5A) (Tryfona *et al.*, 2012; Tan *et al.*, 2013; Shimoda *et al.*, 2014). In addition to these neutral and acidic oligosaccharides, we detected at least six non-assigned oligosaccharides that were susceptible to β-glucuronidase and/or α-l-arabinofuranosidase digestion, indicating that β-glucuronidated or α-l-arabinosylated oligosaccharides, which are also typically found in type II AG, were released in the transgenic plants (Fig. 5B; Supplementary Fig. S3). Because these AG-specific oligosaccharides were not observed in either WT or *Dex::Il3GAL*-*PM* plants with Dex treatment, or in *Dex::Il3GAL* plants without Dex treatment, we conclude that they must have been liberated as a result of the hydrolysis of type II AGs by Il3GAL.

### Tissue disorganization in the hypocotyl

**Fig. 6.**
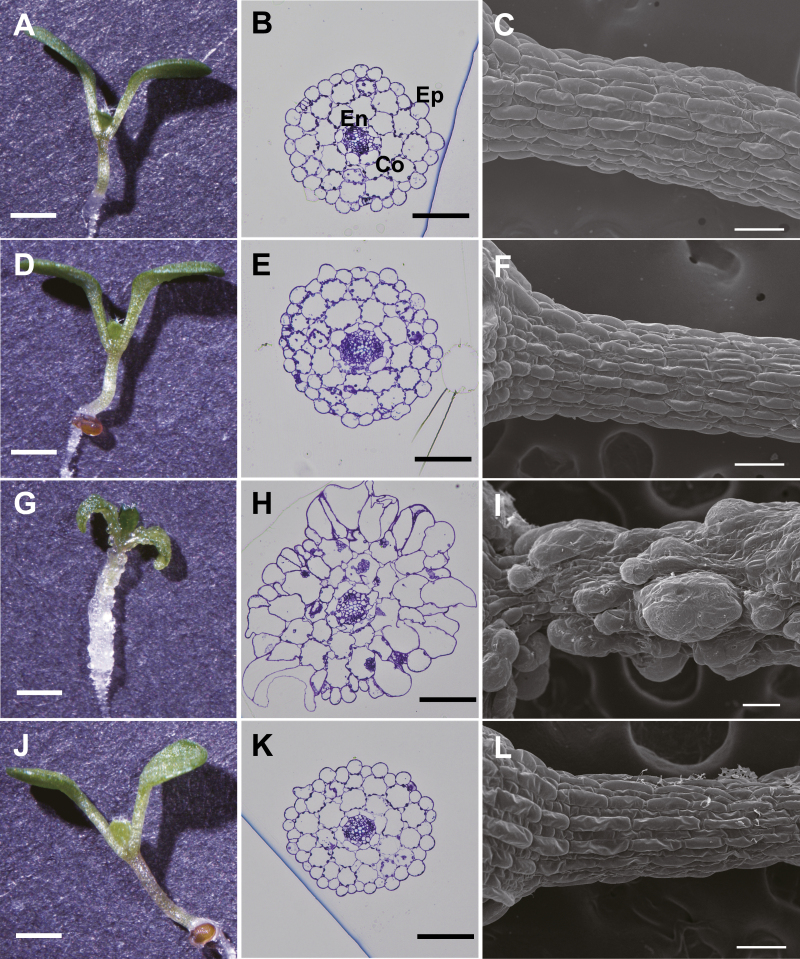
Tissue disorganization in the transgenic plants. WT plants treated with Dex (A–C), *Dex::Il3GAL* plants grown without Dex (D–F), *Dex::Il3GAL* plants treated with Dex(G–I), and *Dex::Il3GAL-PM* plants treated with Dex (J–L) were observed. Plants were grown on MS agar medium containing 0.1 μM Dex for 7 d under continuous light before analysis. (A, D, G, J) Stereoscope images of seedlings. Scale bar=1 mm. Plants treated with different Dex concentrations are shown in Supplementary Fig. S4. (B, E, H, K) Cross-sections of the middle region of the hypocotyl stained with toluidine blue O. Scale bar=100 µm. Ep, epidermis; Co, cortex; En, endodermis. (C, F, I, L) SEM images of hypocotyls. Scale bar=100 μm.

Four out of five lines of *Dex::Il3GAL* plants (#1–#3 and #5) showed abnormal morphology in hypocotyls and cotyledons when grown on MS agar medium containing 0.1–10 μM Dex under light conditions (Fig. 6; Supplementary Fig. S4). Under dark conditions, the elongation of etiolated hypocotyls was also significantly decreased (11–56%) in all lines of the Dex-treated *Dex::Il3GAL* plants (Supplementary Fig. S5). Consistent with the galactanase activity, the abnormal morphology was enhanced by increased concentrations of Dex in the growth medium. Observation by SEM revealed extreme bulging of some epidermal cells of the hypocotyl (Fig. 6I). In the cross-sections of hypocotyls of #2 plants, abnormally large cells were found not only in the epidermis, but also in the cortex and endodermis, resulting in severe tissue disorganization in the hypocotyl, while no abnormally enlarged cells were observed in the vascular tissues (Fig. 6H). Because WT and *Dex::Il3GAL-PM* plants did not show any change under Dex treatment, it can be assumed that the degradation of type II AGs was the cause of these distinctive phenotypes (Fig. 6A–F, J–L). These results therefore indicate that the type II AGs play an important role in regulation of the cell morphology. The differences in appearance and varying extent of disorganization in the phenotypes of transgenic lines may result from different expression patterns and levels of Il3GAL.

### Effect of exogenous application of type II AG oligosaccharides

We considered the possibility that cell bulging and tissue disorganization were caused by accumulation of Gal or oligosaccharides released from type II AGs by the action of Il3GAL. It has been reported that a high concentration of monomeric Gal inhibits cell elongation (Yamamoto *et al.*, 1988; Dörmann and Benning, 1998; Rösti *et al.*, 2007). In order to examine whether the Gal and oligosaccharides produced by the action of Il3GAL caused the growth defects, we prepared a hydrolysate of Arabidopsis AGP by rIl3GAL *in vitro* and applied it to WT plants growing in MS medium. However, the application of hydrolysate did not mimic the tissue disorganization phenotypes of *Dex::Il3GAL* plants (Supplementary Fig. S6). This suggests that the reduced amount of type II AGs, rather than the released oligosaccharides, caused the tissue disorganization.

## Discussion

### Specific degradation of type II AGs *in vivo*

Type II AGs are the second most complex polysaccharides after pectin rhamnogalacturonan II in plants. Type II AGs mainly consist of Gal and l-Ara, but also possess other sugars such as GlcA, 4-*O*-Me-GlcA, l-Fuc, l-Rha, and Xyl, which differ depending on plant species, tissue, and age (Tsumuraya *et al.*, 1988). Heterogeneous carbohydrate structures make functional analysis difficult. In AGPs from radish, wheat (*Triticum aestivum* L.), and pear (*Pyrus communis* L), the length of β-1,6-galactan side chains varies from one to >20 (Tryfona *et al.*, 2010; Shimoda *et al.*, 2014; Tsumuraya *et al.*, 2019). Therefore, in the past, the specific *in vitro* fragmentation of type II AGs into oligosaccharides using microbial hydrolases such as exo-β-1,3-galactanase and endo-β-1,6-galactanase has been an effective approach to obtain structural information (Tryfona *et al*., 2010, 2012; Shimoda *et al.*, 2014; Inaba *et al.*, 2015).

The present study is the first attempt to achieve specific degradation of type II AGs *in vivo*. Il3GAL is quite specific to β-1,3-galactan, as it never acts on glycans including carboxymethyl-curdlan (β-1,3-glucan), β-1,4-galactan, pectic arabinan, and xylan (Tsumuraya *et al.*, 1990; Kotake *et al.*, 2009). Therefore, it is unlikely that Il3GAL induced by Dex hydrolyzed glycans other than type II AGs in *Dex::Il3GAL* plants. We found that in some of these plants, the amount of β-Yariv-reactive type II AGs decreased to as little as 30%. On the other hand, this observation indicates that ~30% of type II AGs remained and were not hydrolyzed by Il3GAL. It is probable that in some tissues the expression of *Il3GAL* gene was not induced by Dex treatment. It is also possible that some of the type II AGs were resistant to hydrolysis by Il3GAL. In a previous study, we have observed that rIl3GAL expressed in *Pichia* yeast had weak activity toward gum arabic (acacia gum), which contains β-1,3-galactans highly substituted with short β-1,6-galactan side chains (Kotake *et al.*, 2009). Type II AGs having such a structure, if present, would not be hydrolyzed *in vivo* by Il3GAL.

### Inhibitory effect of type II AG degradation *in planta*

It has been suggested that type II AGs are implicated in a wide range of physiological processes in plants (Park *et al.*, 2003; Lee *et al.*, 2005). However, most information about the physiological importance of type II AGs so far comes from studies using β-Yariv reagent and/or Arabidopsis knockout lines. Our system instead works by specific and controlled degradation of type II AGs *in vivo*. Several phenotypes we observed, including cell bulging and tissue disorganization, were similar to those of Arabidopsis roots treated with β-Yariv reagent and the knockout *fla4/sos5* mutant under salt stress (Willats and Knox, 1996; Shi *et al.*, 2003), indicating that the Dex treatment caused inhibition of AGP functions in *Dex::Il3GAL* plants. In addition, severe tissue disorganization in the hypocotyl and cotyledons, which has not been reported in AGP mutants under normal conditions, was observed in *Dex::Il3GAL* plants. The reason for this may well be that the drastic degradation of type II AGs occurred not only in surface tissues but also in tissues such as the cortex and endodermis. Indeed, in *Dex::Il3GAL* #2 plants, ~70% of β-Yariv-reactive type II AGs were lost, which should lead to many kinds of AGPs losing their native functions. The present study also shows the practical advantage of the Dex-inducible system, because this system allows controlled analysis of the influence of *Il3GAL* expression, which causes severe and lethal phenotypes when fully unleashed.

If the system presented in this study is to serve as a new tool in the study of the functions of type II AGs and AGPs, we need to consider to what extent the accumulation of Il3GAL protein induced by Dex treatment may have directly affected the phenotypes. Indeed, we observed a dwarf phenotype in one line (#6) of *Dex::Il3GAL-PM* plants out of nine lines (Supplementary Figs S4, S5). However, the severe tissue disorganization occurring in four lines (#1, #2, #3, and #5) of *Dex::Il3GAL* plants was not observed in any lines of *Dex::Il3GAL-PM* plants, suggesting that tissue disorganization resulted from the degradation of type II AGs by Il3GAL, rather than protein accumulation.

### Participation of type II AGs in tissue organization

Plant cell shape is primarily determined by the orientation of cellulose microfibrils. Deposition of cellulose microfibrils involves CMTs, along which cellulose synthase complexes move within the plane of the plasma membrane. It is conceivable that the degradation of type II AGs influences the orientation and/or stabilization of CMTs. In fact, depolymerization and disorganization of CMTs together with cell bulging are caused by treatment with β-Yariv reagent in tobacco cultured cells (Sardar *et al.*, 2006). Moreover, similar disorganization of CMTs and swelling were also reported in Arabidopsis roots treated with β-Yariv reagent for 24 h (Nguema-Ona *et al.*, 2007). To examine the possibility that degradation of type II AGs affects CMTs, CMTs were observed in *Dex::Il3GAL* #2 plants by introducing a gene encoding microtubule-associated protein (MAP) 65-1 fused with green fluorescence protein (GFP) (Lucas *et al.*, 2011; Soga *et al.*, 2018). In a time course experiment, the change in the orientation of CMTs occurred along with that in cell shape 48 h after the Dex treatment (Supplementary Fig. S7). Although the degradation of type II AGs significantly influenced CMTs, the change in CMTs may have been brought about indirectly by the change in cell shape. We note that the change observed in CMTs in *Dex::Il3GAL* #2 plants was milder than that in Arabidopsis mutants impaired in the regulation of CMTs such as *mor1* and *clasp* (Whittington *et al.*, 2001; Ambrose et al., 2007, 2011).

In conclusion, we have developed a novel approach to achieve the specific degradation of type II AGs *in vivo*. Using this system, we were able to show that type II AGs are physiologically important and participate in the organization of hypocotyl and cotyledon tissues. Future studies will aim to identify the carbohydrate structure(s) responsible for these biologically relevant functions of type II AGs. It may well be that specific structures in type II AGs play important roles in cellulose synthesis. Because Il3GAL hydrolyzed the β-1,3-galactan backbone causing drastic overall degradation of type II AGs, the identification of the structures essential for the physiological function of type II AGs was not within the scope of the present study. Further approaches using other hydrolases that specifically act on type II AGs, including endo-β-1,6-galactanase and β-glucuronidase, will help to identify such important carbohydrate structures.

## Supplementary data

Supplementary data are available at *JXB* online.

Table S1. List of primers used in this study.

Fig. S1. Gene constructs for the generation of *Dex::Il3GAL* and *Dex::Il3GAL-PM* plants.

Fig. S2. Dose-dependent increase in galactanase activity.

Fig. S3. The elution positions of standard β-1,6-galactooligo saccharides and MeGlcA-β-1,6-galactooligosaccharides.

Fig. S4. Phenotype of *Dex::Il3GAL* and *Dex::Il3GAL-PM* plants.

Fig. S5. Length of etiolated hypocotyls of *Dex::Il3GAL* and *Dex::Il3GAL-PM* plants.

Fig. S6. Effect of AG oligosaccharides liberated by rIl3GAL on seedlings

Fig. S7. Effects of degradation of type II AGs on CMTs.

eraa236_suppl_Supplementary_Material

## Abbreviations

ABEE: *p*-aminobenzoic acid ethyl ester
Arafase: α-l-arabinofuranosidase from *Aspergillus niger*
AG: arabinogalactan
AGP: arabinogalactan-protein
l-Ara*f*: l-arabinofuranose
AraGal3: l-arabinosyl-β-1,6-galactotriose
CMT: cortical microtubule
Dex: dexamethasone
Gal_2_: β-1,6-galactobiose
Gal_3_: β-1,6-galactotriose
Gal_4_: β-1,6-galactotetraose
GFP: green fluorescence protein
GlcAase: recombinant β-glucuronidase from *Aspergillus niger*
Il3GAL: exo-β-1,3-galactanase from *Irpex lacteus*
IMO: isomaltooligosaccharide
4-*O*-MeGlcA: 4-*O*-methyl-GlcA
MeGlcAGal: MeGlcA-β-1,6-Gal
MeGlcAGal_2_: MeGlcA-β-1,6-Gal_2_
MeGlcAGal_3_: MeGlcA-β-1,6-Gal_3_
MS: Murashige and Skoog
rIl3GAL: recombinant Il3GAL
rIl3GAL-PM: point-mutated rIl3GAL
rNcEn3GAL: recombinant endo-β-1,3-galactanase from *Neurospora crassa*
WT: wild-type.
