# Supplementary material for: Expression of a fungal exo-β-1,3-galactanase in Arabidopsis reveals a role of type II arabinogalactans in the regulation of cell shape
Source: J Exp Bot. 2020 May 29;71(18):5414–24. doi: 10.1093/jxb/eraa236 (PMC7501824; doi:10.1093/jxb/eraa236)
Supplement: eraa236_suppl_Supplementary_Material [file eraa236_suppl_supplementary_material.pdf]

Supplemental information for

Probing the importance of type II arabinogalactan in vivo by expressing a specific fungal exo-β-1,3-galactanase in Arabidopsis

Yoshihisa Yoshimi, Katsuya Hara, Mami Yoshimura, Nobukazu Tanaka, Takumi Higaki, Yoichi Tsumuraya, and Toshihisa Kotake

Toshihisa Kotake
Email: kotake@mail.saitama-u.ac.jp

This PDF file includes:

Table S1
Figures S1 to S7

Table S1. List of primers used in this study.

**Primer**	**Sequence (5' to 3')**
AGP4signal-F+*Xho*I	5'-*CTCGAG*ATTATGGGTTCCAAGATTG-3'
AGP4signal-R+*BamH*I	5'-*GGATCC*AGCGAGTGCTGAAGTGGCGA-3'
Il3GAL-F+*Bam*HI	5'-*GGATCC*GAGACACAGATCGTCTC-3'
Il3GAL-R+*Spe*I	5'-G*ACTAGT*TAGTAGACGATAATCTTGTC-3'
Il3GAL-PM1-F	5'-ATCGTTGCCCGTCCCAAAGT-3'
Il3GAL-PM1-R	5'-GGGACGCGCAACGATGTTGT-3'
Il3GAL-QPCR-F	5'-GCACTGGGTTGCGATCTATT-3'
Il3GAL-QPCR-R	5'-GTTTGCGCCATTATTCAGGT-3'
ACT2-RTP-F	5'-ACCTTGCTGGACGTGACCT-3'
ACT2-RTP-R	5'-CACCAATCGTGATGACTTGC-3'

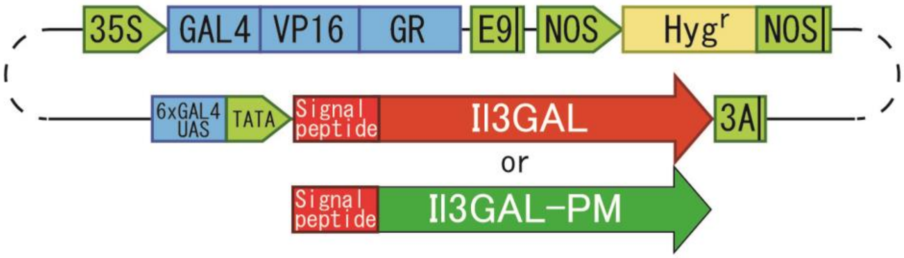

Fig. S1. Gene constructs for the generation of *Dex::Il3GAL* and *Dex::Il3GAL-PM* plants. The *Il3GAL* or *Il3GAL-PM* fragment fused with the signal sequence of Arabidopsis AGP4 (AGP4-SP) was subcloned between the GAL4 binding sequence (*6xGAL4 UAS*) and the pea rbsc 3A terminator in pTA7001. In the transgenic Arabidopsis, a chimeric transcription factor consisting of DNA-binding domain (*GAL4*), transactivating domain from herpes virus (*VP16*), and rat glucocorticoid receptor (*GR*) is constitutively expressed under the cauliflower mosaic virus 35S promoter. By changing the intracellular localization of GAL4-VP16-GR fusion protein from the cytosol to the nucleus, Dex induces the expression of Il3GAL or Il3GAL-PM. pNos, promoter sequence from nopaline synthase gene; tNos, transcription terminator sequence from nopaline synthase gene; Hygr, hygromycin resistance gene; E9, pea rbsc-E9 terminator.

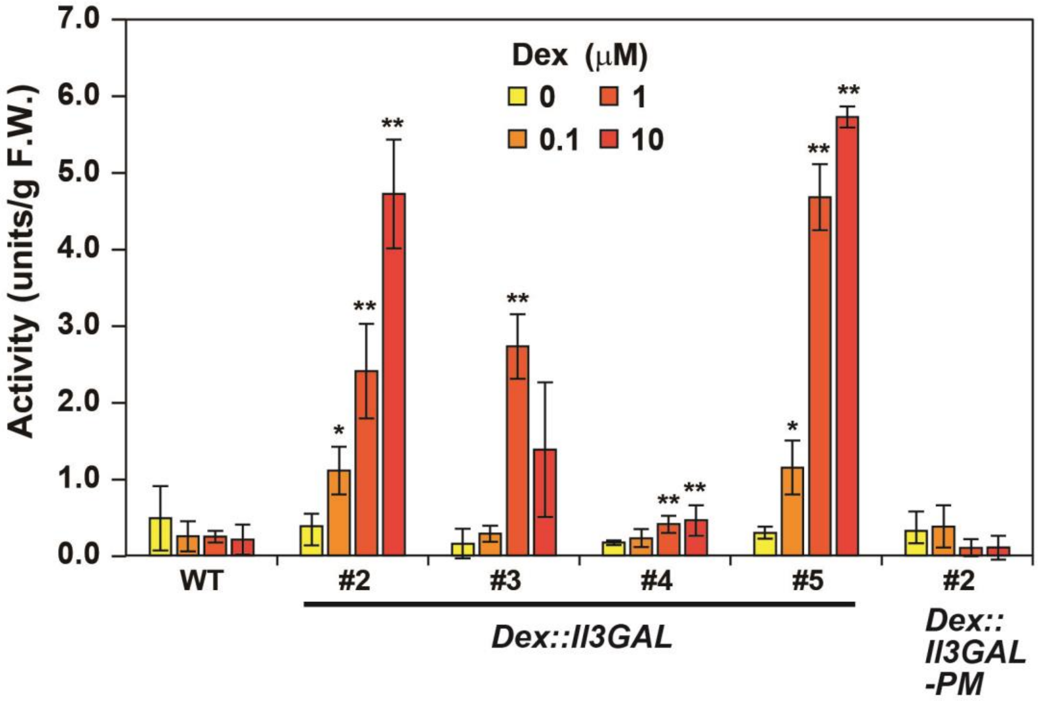

Fig. S2. Dose-dependent increase in galactanase activity. Seedlings of *Dex::Il3GAL* or *Dex::Il3GAL-PM* plants were first grown in the absence of Dex for two weeks and then treated with 0, 0.1, 1, 10 μM Dex for two days. The values shown represent the combined activities of the soluble and wall-bound fractions. Data are mean values with ±SD (n=3 biological replicates). Asterisks indicate significant differences between samples with and without Dex-treatment (Student's t-test, *, P<0.05; **, P<0.01)

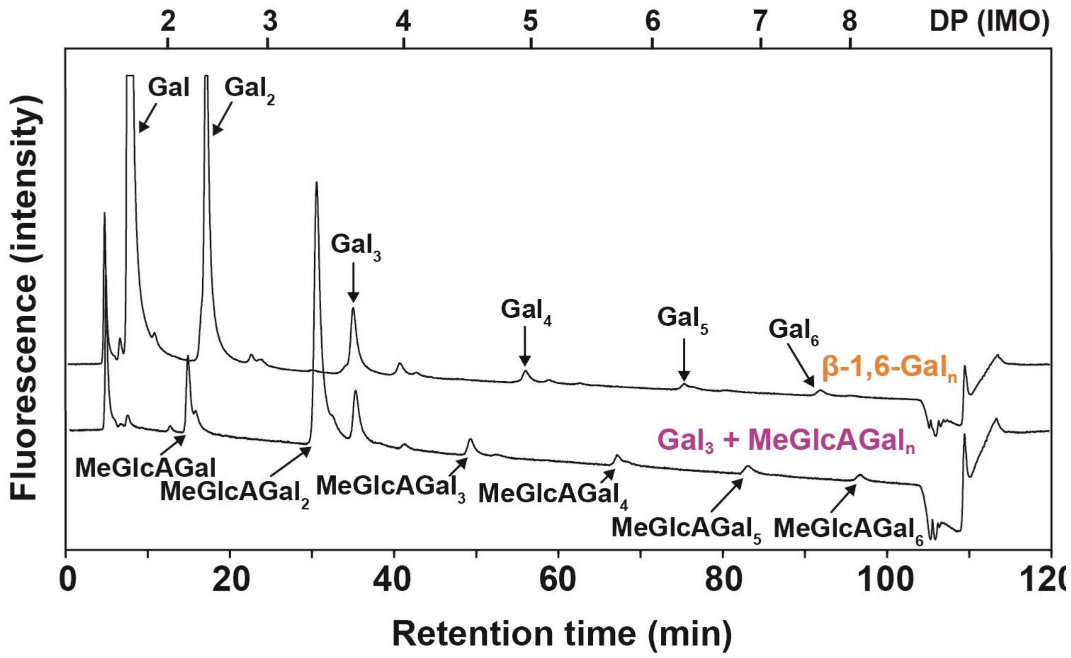

Fig. S3. The elution positions of standard β-1,6-galactooligosaccharides and MeGlcA-β-1,6-galactooligosaccharides. The oligosaccharides were prepared from radish root AGP. The oligosaccharides shown in Figure 5 were assigned based on the elution positions of β-1,6-galactooligosacchrides (β-1,6-Gal_n) and MeGlcA-β-1,6-Gal_n. The elution positions of glucose and IMOs with DP 2-8 are indicated on the top.

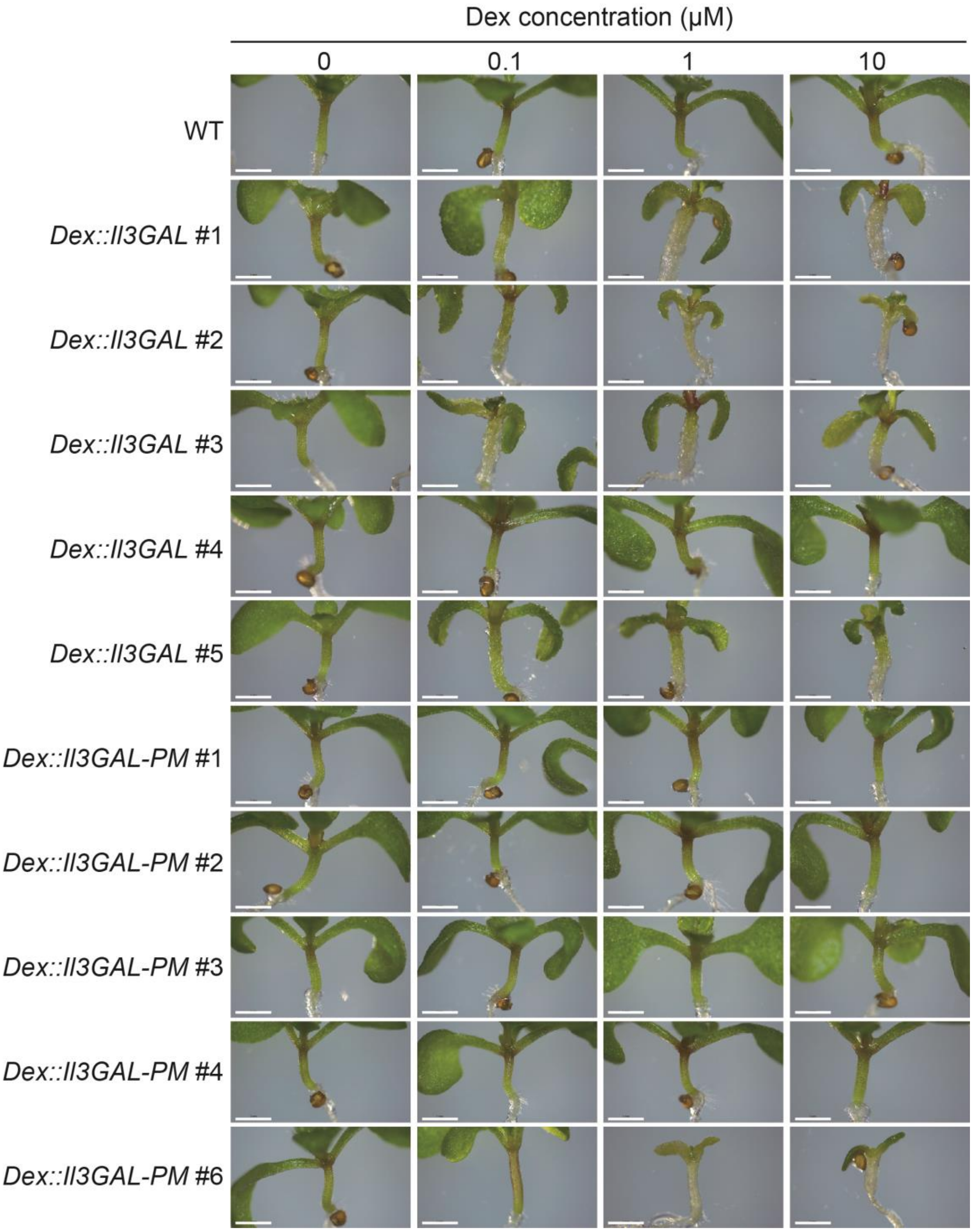

Fig. S4. Phenotype of *Dex::Il3GAL* and *Dex::Il3GAL-PM* plants. Plants were germinated and grown on MS-agar plate containing 0, 0.1, 1 and 10 μM Dex under continuous light for 7 days. Stereoscopic microscopy revealed tissue disorganization in the hypocotyl and cotyledons of *Dex::Il3GAL* #1, 2, 3, and 5 plants. Scale bar = 1 mm.

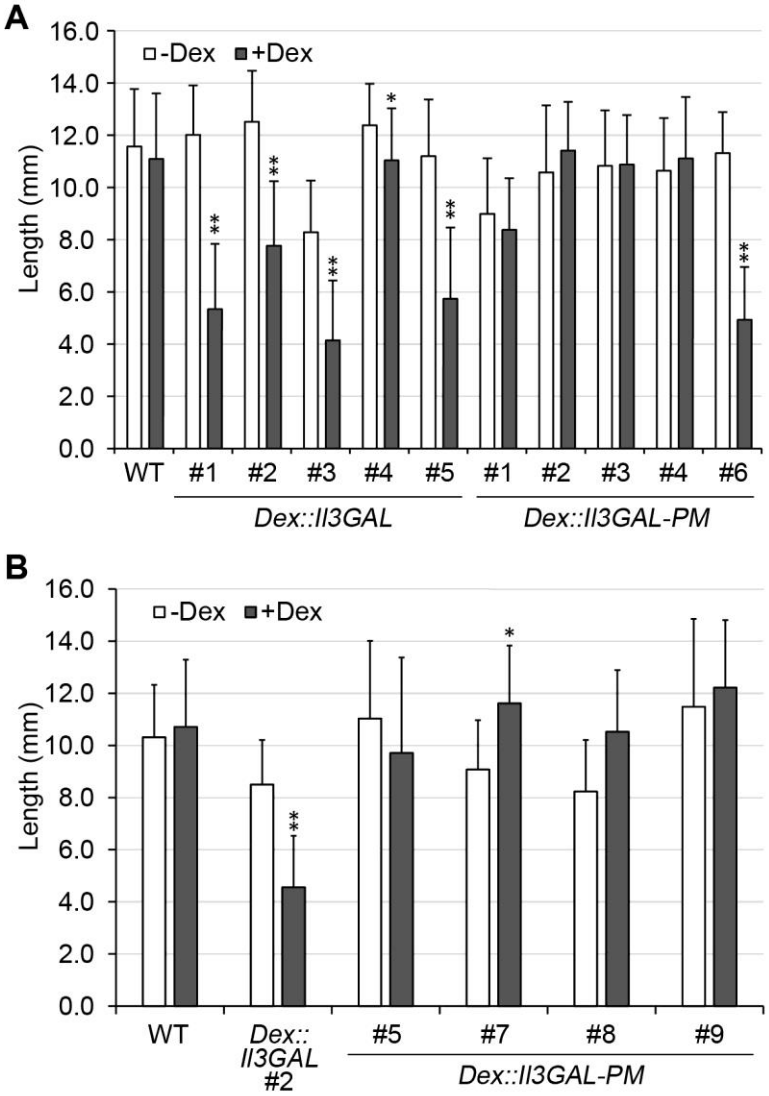

Fig. S5. Length of etiolated hypocotyls of *Dex::Il3GAL* and *Dex::Il3GAL-PM* plants. (A,B) Plants were grown on MS-agar plate with and without 0.1 µM Dex. The length of five-day-old hypocotyls was measured using ImageJ software. Reduced elongation observed in *Dex::Il3GAL-PM* #6 suggests that the expression of Il3GAL and Il3GAL-PM proteins causes stress to a certain extent. The tissue disorganization that occurred in *Dex::Il3GAL* #1, 2, 3, and 5 plants, was not observed in *Dex::Il3GAL-PM* plants. The results shown in (A) and (B) are derived from experiments performed independently. The data show mean values with ±SD. n = 21~76 (A), 15~22 (B). Asterisks indicate significant differences between plants with and without Dex treatment (Student's t-test, *, $P<0.05$; **, $P<0.01$).

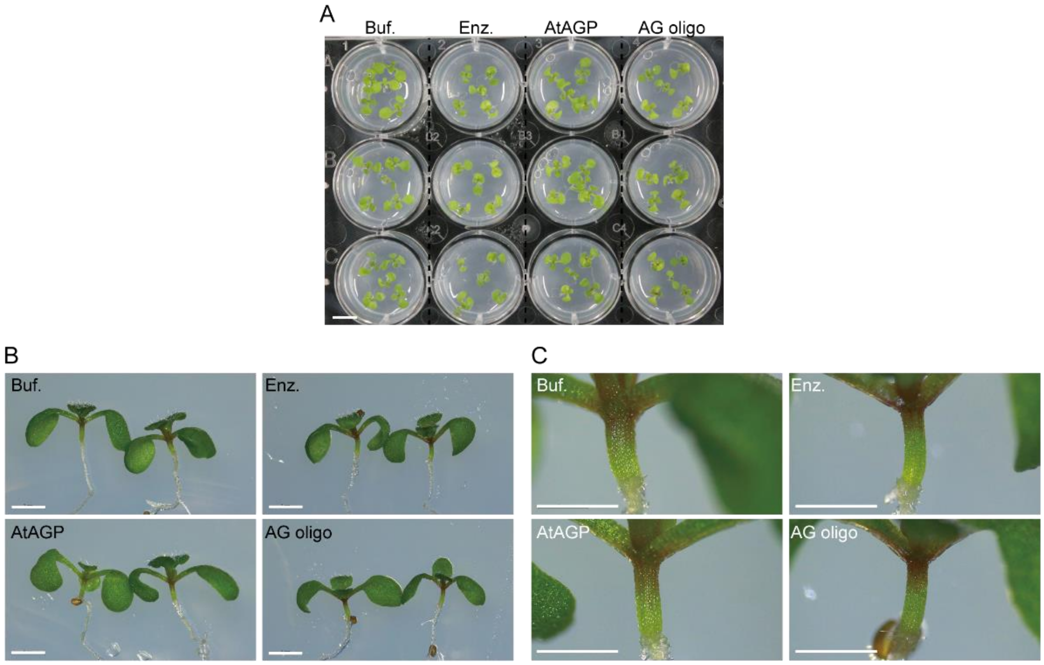

Fig. S6. Effect of AG oligosaccharides liberated by rII3GAL on seedlings. (A) Treatment of wild-type plants with AG oligosaccharides. Wild-type seeds were sown in liquid MS medium and seedlings grown for seven days under continuous light. The MS medium contained 20 mM MES-KOH buffer pH 5.5 (Buf.), 1.4 μg/mL rII3GAL (100 mU) (Enz.), 0.1 mg/mL of AGP purified from Arabidopsis (AtAGP), or 0.1 mg/mL of oligosaccharides prepared from Arabidopsis AGP by hydrolysis with rII3GAL (AG oligo). (B) Stereoscope images of treated seedlings. Scale bar = 2 mm. (C) Observations at high magnification. Scale bar = 1 mm. The treatment with oligosaccharides did not cause tissue disorganization.

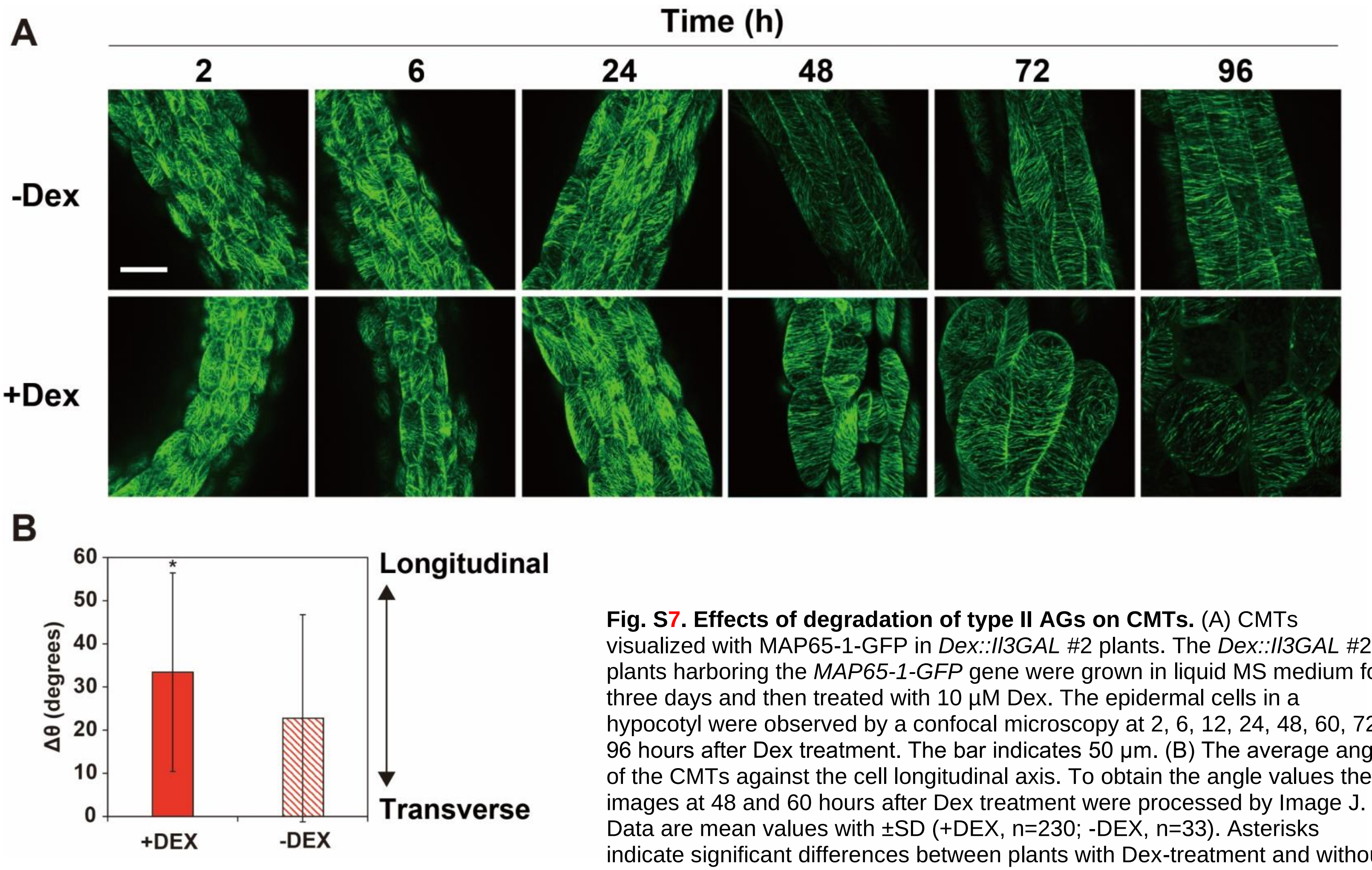

Fig. S7. Effects of degradation of type II AGs on CMTs. (A) CMTs visualized with MAP65-1-GFP in *Dex::Il3GAL* #2 plants. The *Dex::Il3GAL* #2 plants harboring the *MAP65-1-GFP* gene were grown in liquid MS medium for three days and then treated with 10 µM Dex. The epidermal cells in a hypocotyl were observed by a confocal microscopy at 2, 6, 12, 24, 48, 60, 72, 96 hours after Dex treatment. The bar indicates 50 µm. (B) The average angle of the CMTs against the cell longitudinal axis. To obtain the angle values the images at 48 and 60 hours after Dex treatment were processed by Image J. Data are mean values with ±SD (+DEX, n=230; -DEX, n=33). Asterisks indicate significant differences between plants with Dex-treatment and without Dex treatment (Mann–Whitney U test, *, P<0.01).
